# Transcriptomic data indicating differential expressed genes between HIV-1 Associated Nephropathy (HIVAN) mouse model (Tg26) and wildtype mice

**DOI:** 10.1016/j.dib.2020.105562

**Published:** 2020-04-17

**Authors:** V. Praveen Chakravarthi, Sireesha Yerrathota, Priyanka Radadiya, Clark Bloomer, Sumedha Gunewardhana, Madhulika Sharma

**Affiliations:** a Department of Internal Medicine, University of Kansas Medical Center, Kansas City, KS 66160, United States; b Department of Pathology and Laboratory Medicine, University of Kansas Medical Center, Kansas City, KS, United States; c Department of Molecular and Integrative Physiology, University of Kansas Medical Center, Kansas City, KS, United States; d Department of the Jared Grantham Kidney Institute, University of Kansas Medical Center, Kansas City, KS, United States

**Keywords:** HIVAN, Chronic kidney disease, Inflammation, Tg26

## Abstract

Tg26 mice are robust models of human immunodeficiency virus 1 associated nephropathy (HIVAN). These mice are useful for HIVAN pathology analysis, and recent studies suggest that the Tg26 mouse model is an excellent model of other chronic kidney diseases. We performed RNA seq analysis of differential gene expression in the kidneys of Tg26 mice. Kidneys were collected from Tg26 mice and wildtype (WT) littermates at 3 months of age. The raw data were analyzed for differential gene expression using a negative binomial generalized linear model in the DeSeq2 software package. We used *P*-Value ≤0.05 and an absolute fold change of 1.5 to identify top 50 upregulated and top 50 downregulated differentially expressed genes between the WT and Tg26 mice. As expected inflammatory genes were among the top differentially regulated genes. Our data provides yet another level of information to help gain a more comprehensive understanding of disease progression and identify potential drug targets for HIVAN and chronic kidney diseases.

Specifications Table

**Table:** 

Subject	Biology
Specific subject area	Nephrology and HIV
Type of data	RNA-sequencing data table in excel format as supplementary
How data were acquired	Illumina NovaSeq 6000
Data format	Raw transcript(gene) abundant estimates generated by RSEM [1]. Normalized gene expression values given as median of ratios generated by the DESeq2 package [2]. Differential gene expression estimates of Tg26 *vs.* WT calculated using the DESeq2 package [2].
Parameters for data collection	Data collected is representative of 6 Tg26 mice (3 males and 3 females) and 4 WT mice (2 males and 2 females) at 3 months of age. At 3 months most Tg26 mice have the kidney phenotype.
Description of data collection	Kidneys from WT and Tg26 mice were used for isolation of RNA using (Trizol). Library construction and sequencing were done in the genomics core as described in material and methods.
Data source location	A basic Science Laboratory at the University of Kansas Center, Kansas City, USA
Data accessibility	Repository name: [Sequence Read Archive (SRA)]
	Data identification number: [PRJNA578136]
	Direct URL to data: https://trace.ncbi.nlm.nih.gov/Traces/study/?acc=PRJNA578136

## Value of the data

• The data are useful for identifying the candidate genes that are regulated by expression of the HIV-1 proteins.
• These data will be of benefit to researchers who are interested specifically in the field of HIV Associated Nephropathy and can also be useful to HIV researchers in general.
• The data opens avenues for further testing these differential expressed genes for therapeutic options in chronic kidney disease and HIV associated nephropathy.
• The data can be used for discovery of novel targets that regulate HIV-1.
• The data adds to the existing knowledge regarding Tg26 mouse model of HIVAN.

## 1 Data description

In Tg26 mice, the HIV-1 LTR promoter harbors seven of the nine HIV-1 genes and regulate their expression. The deletion of a portion of *gag* and *pol* gene renders this mouse model non-infectious [3]. In this article we report the differences in the transcriptomic profile, derived from RNA-sequencing, of the kidneys obtained from Tg26 mice and wildtype littermates. The data comprises both male and female mice. The WT group consists of 2 males and 2 females (*n* = 4). The Tg26 group consists of 3 males and 3 females (*n* = 6). The accession codes for these samples deposited in the SRA database are, two WT males (SRR10302276, SRR10302275), two WT females(SRR10302274, SRR10302273), three Tg26 males (SRR10302272, SRR10302271, SRR10302270) and three Tg26 females (SRR10302269, SRR10302268, SRR10302267). The differential expression is presented as top 50 downregulated and top 50 upregulated genes in Tg26 mice verses WT mice and are presented as supplementary data. The list of differentially expressed genes is listed as absolute fold change ≥ 1.5,  *p* ≤ 0.05) as described [4].

## 2 Experimental design, materials, and methods

### 2.1 WT and Tg26 mice

The Tg26 mouse is a widely studied model of HIVAN in which all HIV-1 proteins except gag and pol are expressed [3,5]. Homozygous Tg26 mice die early within one month. The heterozygous Tg26 mice (referred to as Tg26 in this manuscript) replicate HIVAN pathology, which is mainly due to the transgene expression in the kidneys [6]. Wild type (WT) and Tg26 mice (Jackson laboratories) used, were raised in accordance with the guidelines of the Guide for the Care and Use of Laboratory Animals of the National Institutes of Health (NIH). All the mice used were housed in micro-isolator cages, pathogen-free conditions, on air-filtered, ventilated rack. The protocols used in research were approved by the Institutional Animal Care and Use Committee (IACUC) of the University of Kansas Medical Center (Kansas City, KS). Tg26 mice and WT mice were bred in pure FVB background. Three males and three females comprised of the Tg26 group and the WT group comprised of two females and two males, all 3 months of age. Genotyping was performed as described before using polymerase chain reaction and primers described before [5].

### 2.2 Sample collection and processing

Mice were euthanized, at 3 months for collection of renal tissue following perfusion with ice cold PBS to get rid of the blood cells. Isolated kidneys were snap frozen in liquid nitrogen and stored at −80 °C until processed for RNA isolation as described previously [7]. Total RNA was extracted using Trizol (Fisher Scientific) using manufacturer's protocol. RNA integrity was determined on the Agilent Bioanalyzer 2100 (Agilent Technologies, Santa Clara, CA) using the RNA6000 Nano assay kit VII (Agilent Technologies).

Global transcriptomic analysis, preparation of RNA-seq libraries, sequencing of cDNA libraries was performed using Affymetrix Clariom D (Thermo Fisher Scientific) array at the Genomics Core Facility of the University of Kansas Medical Center. Briefly, RNA-seq libraries were prepared using a TruSeq Stranded mRNA kit (Illumina) following the manufacturer's instructions. Briefly, mRNA was enriched from total RNA (500 ng per sample) by oligo-dT magnetic beads, purified and chemically fragmented. The first strand of cDNA was synthesized using random hexamer primers and reverse transcriptase. Double stranded cDNA (ds cDNA) was generated by removing the RNA template and synthesizing a replacement strand, incorporating dUTP in place of dTTP. AMPure XP (Beckman Coulter) beads were used to purify ds cDNAs from the second strand reaction mix. The cDNA ends were blunted and a poly (A) tail was added to the 3′ ends. Ligation of indexing adaptors (Illumina) was done and the suitable DNA fragments were selected for PCR amplification using 15 cycles.

### 2.3 RNA-seq data analyses

RNA-Sequencing was performed at a strand specific 100 cycle paired-end resolution, in an illumina NovaSeq 6000 sequencing machine (Illumina, San Diego, CA). Samples from six Tg26 (3 female and 3 male) and four WT (2 female and 2 male) mice were sequenced for this study. Sequencing generated between 28.9 and 33.7 million reads per sample. The read quality was assessed using the FastQC software [8]. On average, the per sequence quality score measured in the Phred quality scale was above 32 for all the samples. The sequenced reads were mapped to both the mouse genome (GRCm38.rel97) and the HIV-1 virus genome (RefSeq: GCF_000864765.1) using the STAR software [9], version 2.6.1c. Between 91.3% and 96.0% of the sequenced reads mapped to the reference genome, resulting in between 27.1 and 32.0 million mapped reads per sample, of which on average 85.4% were uniquely mapped reads.

Transcript abundance estimates were calculated using the RSEM [1] (version 1.3.0) software. Expression normalization and differential gene expression calculations were performed in DESeq2 [2] (version 1.26.0) to identify statistically significant differentially expressed genes. DESeq2 [2] employs a negative binomial generalized linear model (NB-GLM) for statistical calculations. The DESeq2 package implements advance statistical methods to estimate gene-specific biological variation under minimal levels of biological replication. The RNA composition in each sample was normalized in DESeq2 using the Relative Log Expression (RLE) normalization method. The significance *p*-values were adjusted for multiple hypotheses testing by the Benjamini and Hochberg method [10] establishing a false discovery rate (FDR) for each gene.

### 2.4 Accession code

The raw RNA sequencing data was submitted to sequence Read Archive (SRA). The Data identification number is PRJNA578136. The link provided below can be accessed for raw data.

https://trace.ncbi.nlm.nih.gov/Traces/study/?acc=PRJNA578136

### 2.5 Statistical analysis

The DESeq2 software package was used to obtain the statistically significant differentially expressed genes between Tg26 and WT mice as described above. Genes with an absolute fold difference of 1.5 or greater and *p*-value less than or equal to 0.05 were considered significantly differentially expressed.

## Appendix Supplementary materials

Image, application 1
